# The influence of left bundle branch block on myocardial T1 mapping

**DOI:** 10.1038/s41598-024-55821-z

**Published:** 2024-03-05

**Authors:** Antonia Petersen, Sebastian Niko Nagel, Bernd Hamm, Thomas Elgeti, Lars-Arne Schaafs

**Affiliations:** 1 Department of Radiology, Charité—Universitätsmedizin Berlin, corporate member of Freie Universität Berlin and Humboldt-Universität zu Berlin, Hindenburgdamm 30, 12203 Berlin, Germany; 2 Academic Department of Diagnostic and Interventional Radiology and Paediatric Radiology, Protestant Hospital of the Bethel Foundation, Bielefeld University, Medical School and University Medical Center East Westphalia-Lippe, Burgsteig 13, 33617 Bielefeld, Germany

**Keywords:** Cardiology, Cardiovascular diseases

## Abstract

Tissue characterisation using T1 mapping has become an established magnetic resonance imaging (MRI) technique to detect myocardial diseases. This retrospective study aimed to determine the influence of left bundle branch block (LBBB) on T1 mapping at 1.5 T. Datasets of 36 patients with LBBB and 27 healthy controls with T1 mapping (Modified Look-Locker inversion-recovery (MOLLI), 5(3)3 sampling) were included. T1 relaxation times were determined on mid-cavity short-axis images. R^2^ maps were generated as a pixel-wise indicator for the goodness of the fit of T1 maps. R^2^ values were significantly lower in patients with LBBB than in healthy controls (whole myocardium/septum, 0.997, IQR, 0.00 vs. 0.998, IQR, 0.00; p = 0.008/0.998, IQR, 0.00 vs. 0.999, IQR, 0.00; p = 0.027). Manual correction of semi-automated evaluation tended to improve R^2^ values but not significantly. Strain analysis was performed and the systolic dyssynchrony index (SDI_global_) was calculated as a measure for left ventricular dyssynchrony. While MRI is generally prone to artefacts, lower goodness of the fit in LBBB may be mainly attributable to asynchronous contraction. Therefore, careful checking of the source data and, if necessary, manual post-processing is important. New techniques might improve the goodness of the fit of T1 mapping by reducing sampling in the motion prone diastole of LBBB patients.

## Introduction

Left bundle branch block (LBBB) is associated with a higher cardiovascular mortality while its aetiology is manifold. Its prevalence is high and increases with age^1,2^. A LBBB is characterised by a delay in the conduction of left ventricular excitation and thus a delayed contraction of the left ventricle, which leads to typical schemes of dyssynchrony such as “septal flash” (a rapid early systolic deflection of the septum towards the left ventricle) or “apical rocking” (rocking movement of the apex following the contraction of the free left ventricular wall)^3^. Cardiac magnetic resonance imaging (MRI) can help to diagnose underlying structural diseases while also allowing evaluation of myocardial remodeling through the LBBB itself^4^. Cardiac remodeling is associated with altered tissue composition and an increase in fibrosis^5^. In addition to late gadolinium enhancement (LGE) imaging, which is particularly useful for visualising focal areas of fibrosis, tissue characterisation using T1 mapping has become increasingly relevant for detecting and quantifying diffuse myocardial disease^6–10^. Commonly used acquisition schemes for T1 mapping utilise Look-Locker methods and rely on inversion-recovery sequences with several single-shot acquisitions at different inversion times in stand-still diastole to achieve the most congruent position of the myocardium at each inversion time as a prerequisite for pixel-wise mapping^11–13^. While cardiac MRI is generally prone to artefacts due to incorrect triggering or patient movement, little is known about possible effects of conduction abnormalities, such as LBBB, on myocardial T1 measurements^14,15^. Therefore, the aim of the present study is to determine the influence of LBBB on T1 mapping^16^.

## Methods

### Study population

This is an internal review board (IRB)-approved study (application number: EA4/192/21), that conforms to the Declaration of Helsinki. Due to the retrospective nature of the study, the need of informed consent was waived by the IRB of the Charité—Universitätsmedizin Berlin (Ethikkommission Charité—Universitätsmedizin Berlin, Charitéplatz 1, 10117, Berlin). Inclusion criteria were an age of at least 18 and availability of a complete cardiac MRI dataset. All methods were performed in accordance with the relevant guidelines and regulations. A complete cardiac MRI dataset included CINE and LGE imaging in long and short axes as well as unenhanced T1 mapping sequences. All patients with LBBB or left anterior or left posterior hemiblock at the time of an in-patient stay or at the time of image acquisition in an out-patient setting in our hospital from 2016 to 2022 were included in the study. The diagnosis of LBBB or left anterior or left posterior hemiblock was made according to the 2021 European Society of Cardiology criteria, i.e., when the ECG showed a widened Q wave, R wave, S wave (QRS) complex of > 120 ms and any other ECG characteristics of LBBB^2^. Patients without evidence of a conduction delay in whom no cardiac disease was diagnosed on cardiac MRI or further follow-up served as the control group. In these patients, cardiac MRI had been performed mostly because of non-specific thoracic symptoms or suspected myocarditis, which was then ruled out. Clinical information such as cardiovascular risk factors, pre-existing cardiovascular conditions and ECG-characteristics was obtained from the patients' records.

### Image acquisition

All examinations were performed on the same 1.5 T MRI system (Magnetom Aera, Siemens Healthineers, Erlangen, Germany). At the time of image acquisition, all patients were in sinus rhythm. After acquisition of localisers, double-angulated long-axis (2, 3, 4-chamber) and contiguous short-axis slices from the level of the mitral valve to the left ventricular apex (typical parameters: TR 34 ms, TE 1.29 ms, flip angle 5°, in plane resolution 1.7 × 1.7 mm, slice thickness of 5 mm for long-axis acquisition, 8 mm with 2 mm interslice gap for short-axis acquisition) were acquired using a retrospectively gated 2D steady-state free precession (SSFP) pulse sequence. Reconstructed temporal resolution was between 34 and 44 ms^17,18^. Standard LGE imaging was performed 10–12 min after administration of 0.15 mmol/kg gadobutrol (Gadovist®, Bayer AG, Leverkusen, Germany) using a phase-sensitive inversion-recovery (PSIR)-technique. T1 mapping was accomplished using a Modified Look-Locker sequence with 5(3)3 sampling in short axis, covering the whole left ventricle from base to apex in five slices (slice thickness 8 mm, 16 mm interslice gap, TR 378 ms, TE 1.18 ms, flip angle 35°, bandwith 1085 Hz/Px, voxel size 2 × 2 × 8 mm, FOV 328 mm × 384 mm, matrix 164 × 192, iPAT factor (GRAPPA) 2, partial Fourier imaging factor 7/8, similar to protocols from the literature^19^. A non-rigid motion correction was applied scanner-side (Software version: Siemens Healthineers Numaris XA30) to compensate for motion of the diaphragm.

### Post-processing of T1 mapping data/Image analysis

**Figure 1:**
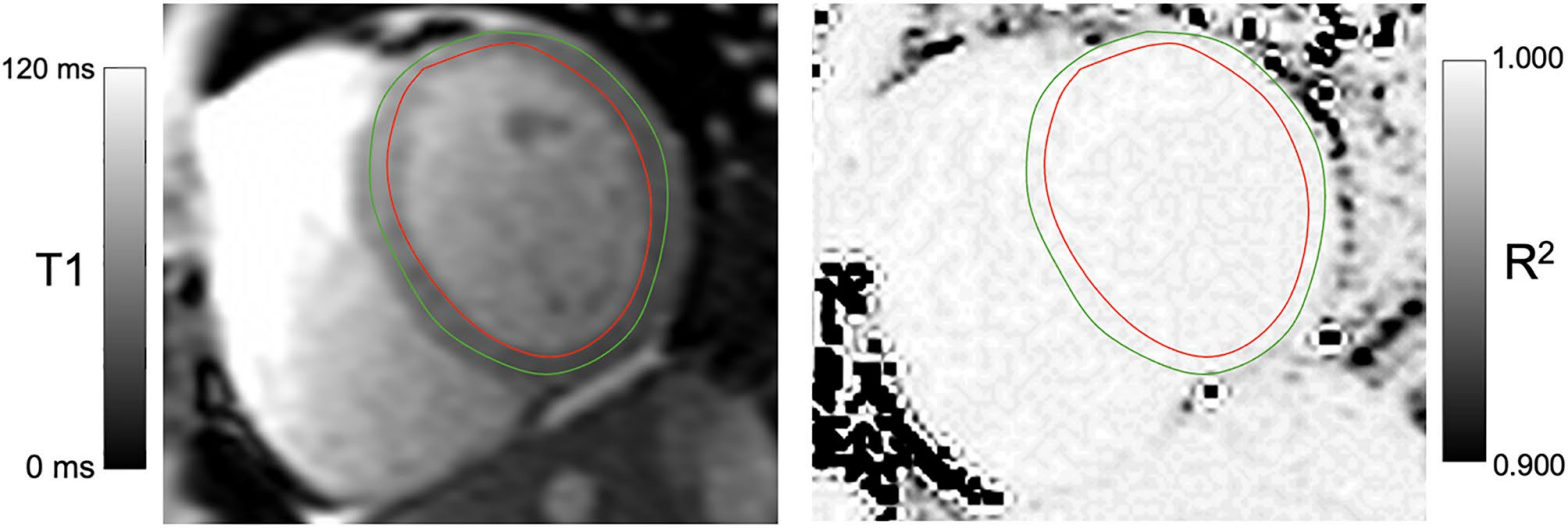
Example of T1 mapping with mid-ventricular epi- and endomyocardial contours on the left and the corresponding R^2^ map on the right. Brighter pixels on the R^2^ map indicate a better goodness-of-the-fit. Areas with T1 values with a poor fit to the T1 recovery curve are displayed as darker pixels.

Prior to further analysis, the entire MRI dataset and in particular T1 mapping sequences were checked for artefacts, such as severe motion artefacts or off-resonance artefacts, and—if necessary—excluded from further analysis. Post-processing of T1 mapping data was carried out using cvi42® [Release 5.14, Circle Cardiovascular Imaging, Calgary, Canada). Epi- and endocardial contours were placed on mid-cavity short-axis images to extract T1 values of the whole myocardium. Additionally, a region of interest (ROI) was placed within the mid-cavity septum to extract septal T1 values (Fig. 1). All contours and ROIs were manually placed on the first shot of the sequence and then semi-automatically forwarded over the remaining single-shot acquisitions. In a second step R^2^ maps were generated as a pixel-wise quality indicator for goodness-of-the-T1 fit (Fig. 1). The goodness of the T1 curve fit might be impacted by the “precision” of the eight individual acquisitions with varying TI that are needed for generation of a T1 map.

As a subanalysis T1 mapping including R^2^ map generation was repeated in patients with LBBB (n = 36) by one reader, with manually placing the epi- and endocardial contours on the first shot of the sequence and correcting them manually in the remaining single-shot acquisitions where it was deemed necessary after semi-automatically forwarding. Four-chamber CINE sequences were analysed for the presence of septal flash and apical rocking. LGE images were evaluated for the presence and distribution of LGE. MRI datasets were evaluated independently by one board-certified radiologist with nine (*BLINDED*) and one resident radiologist (*BLINDED*) with three years of experience in cardiovascular imaging who were blinded to the health status.

### Strain analysis

In a subgroup of patients with LBBB (n = 22) and controls (n = 27), additional strain analysis was performed^20–22^. Summarized, semi-automatically circumferential strain was analyzed using cvi42® [Release 5.13.5 (2190), Circle Cardiovascular Imaging, Calgary, Canada]. When necessary, endo- and epicardial contours had been manually corrected after checking each dataset by two experienced readers in consensus. Global systolic dyssynchrony index (SDI_global_) was calculated as an index of dyssynchronous contraction of the left ventricle (LV) as described previously^21^.

### Statistical analysis

Inter-rater agreement was determined using the intra-class correlation coefficient (two-way random, absolute agreement). Age, QRS width, heart rate, left ventricular function parameters as well as derived values for T1, R^2^ and SDI_global_ were tested for normal distribution using the Shapiro-Wilk test. For normally distributed parameters, due to the different group size a two-sided Welch`s t-test was conducted; this was the case for the age, QRS width, heart rate, left ventricular function parameters and T1 values. If normal distribution could not be assumed, non-parametric testing was performed with the Mann-Whitney-U-test, which was the case for the derived values for R^2^ and SDI_global_. Multiple comparisons were corrected for using the Bonferroni-Holm method. A chi-square test was conducted between LBBB/controls and the clinical characteristics hypertension, smoking, dyslipidaemia and obesity. The exact Fischer test was conducted between LBBB/controls and the clinical characteristics with a cell frequency < 5; these were LGE ischemic, LGE non-ischemic, septal flash, apical rocking, coronary heart disease, non-ischemic cardiomyopathy and diabetes mellitus. A paired-samples sign test was used to compare the semi-automated and manual evaluation in patients with LBBB. The coefficient of variation of the T1 values (SD/mean) was used as an indicator of daily variability of the measurements that can be compared with the literature. The correlation between R^2^ values and clinical characteristics as well as SDI_global_ was calculated using Spearman`s rank correlation coefficient. A p-value < 0.05 was considered statistically significant.

## Results

### Demographics and clinical patient characteristics

Cardiac MRI datasets with T1 mapping from 36 patients with LBBB (11 female) and 27 healthy controls (8 female) were analysed. Demographic and clinical data as well as image-based parameters are compiled in Table 1. Apical rocking was found in 23 of 36 patients, septal flash in 20 patients, both together in 18 of 36 patients. In the control group, there was no LGE, and neither septal flash nor apical rocking was observed.

**Table 1:** Demographics, clinical characteristics and image-based parameters of patients and controls.

Parameters	Patients	Controls	Level of significance
n	36	27	
Age (years)	61 (16)	29 (6)	p < 0.001*
Female/male	11/25	8/19	p = 0.937
Heart rate (beats per minute)	68 (11)	70 (13)	p = 0.625
QRS Width (ms)	153 (22)	91 (8)	p < 0.001*
LVEDV (ml)	224 (71)	147 (35)	p < 0.001*
LVESV (ml)	143 (70)	48 (17)	p < 0.001*
LVSV (ml)	82 (29)	100 (23)	p = 0.009*
LVEF (%)	39 (15)	68 (6)	p < 0.001*
LV myocardial mass (g/m^2^)	77 (28)	61 (14)	p < 0.001*
LGE ischemic	12 (33%)	0	p < 0.001*
LGE non-ischemic	6 (17%)	0	p = 0.033*
Septal flash	20 (56%)	0	p < 0.001*
Apical rocking	23 (64%)	0	p < 0.001*
Coronary heart disease	6 (17%)	0	p = 0.033*
Non-ischemic cardiomyopathy	18 (50%)	0	p < 0.001*
Hypertension	13 (36%)	7 (26%)	p = 0.390
Smoking	2 (6%)	5 (19%)	p = 0.105
Dyslipidaemia	9 (25%)	4 (11%)	p = 0.165
Obesity	3 (8%)	3 (15%)	p = 0.418
Diabetes mellitus	5 (14%)	0	p = 0.065

Continuous parameters are given as mean (SD). Categorial parameters are given as frequency (percentage). An asterisk (*) indicates a statistically significant difference.

### T1 mapping and Strain analysis

**Figure 2:**
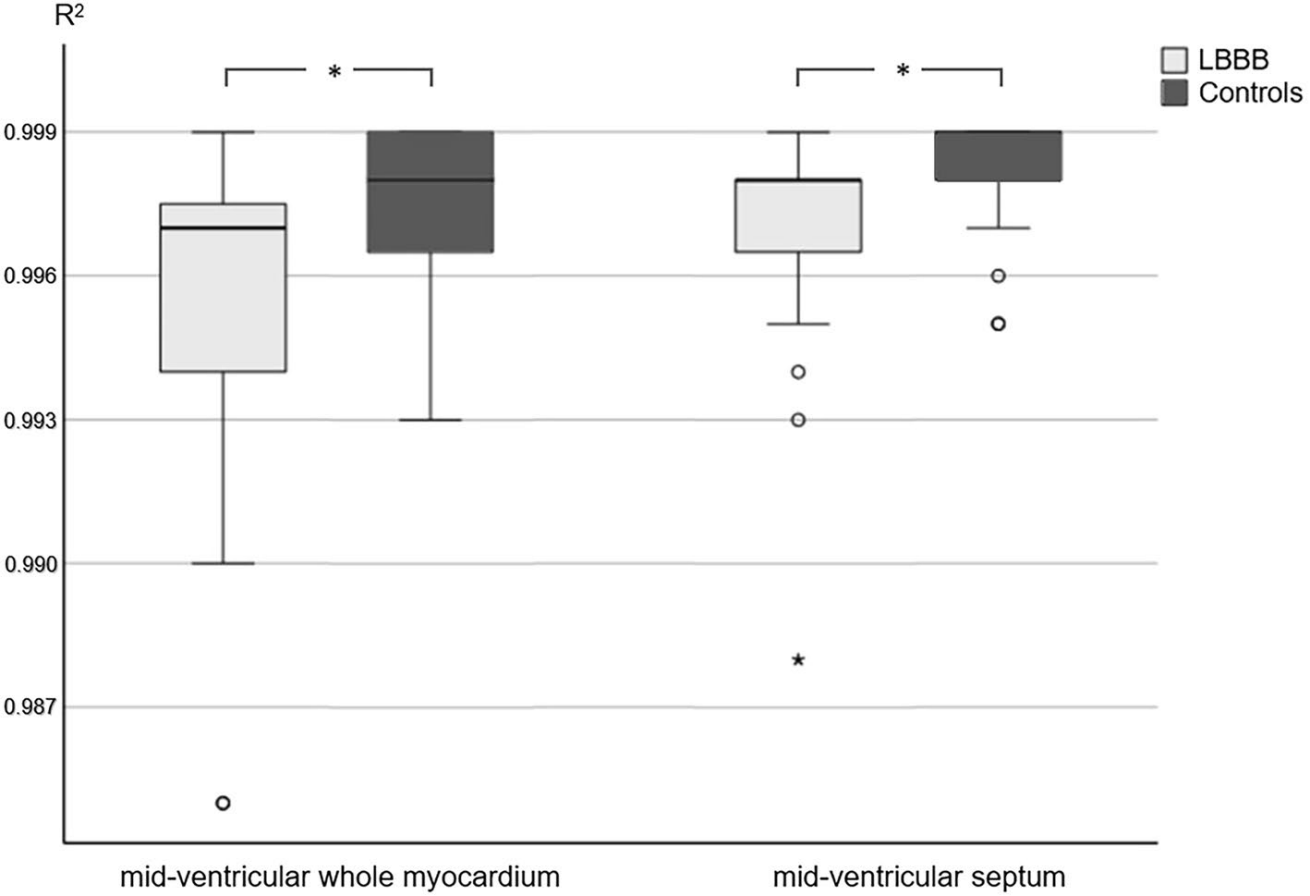
Comparison of R^2^ values in patients with LBBB and controls in the whole mid-ventricular myocardium and the mid-ventricular septum after semi-automatic post processing. An asterisk (*) indicates a statistically significant difference (p < 0.05).

**Figure 3:**
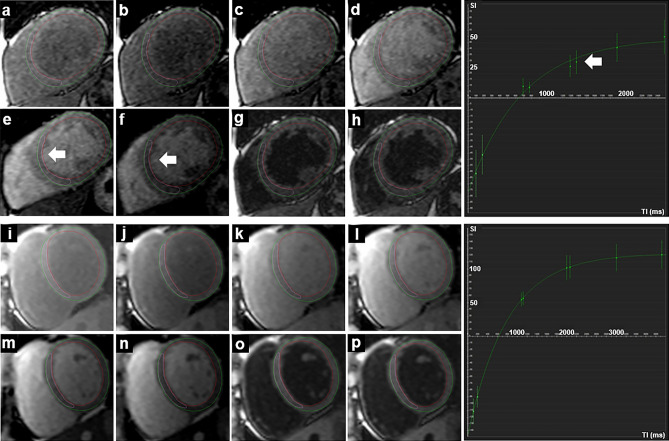
Single-shot acquisitions in T1 mapping (modified Look-Locker sequence) of a patient with LBBB in the upper two rows (**a**–**h**, T1 _whole myocardium_ 1012 ms, R^2^_whole myocardium_ 0.996) and of a healthy control in the lower two rows (**i**–**p**, T1 _whole myocardium_ 972 ms, R^2^_whole myocardium_ 0.999). The respective fitting curve of the whole myocardium is shown to the right, x-axis shows the TI in milliseconds (ms), y-axis shows the signal intensity. The white arrows indicate the deviation of the position of the septum during the eight sequential acquisitions and the resulting poorer fitting to the curve.

Data of the T1 mapping analysis is summarized in Table 2. Measured T1 values of the whole myocardium were significantly higher in patients with LBBB than in the control group, however mean T1 values of the whole myocardium as well as the septal T1 values were within the institute's internal, scanner-specific normal range of 913–1029 ms. R^2^ maps of patients with LBBB showed a poorer goodness-of-the-T1 fit and corresponding significantly lower R^2^ values within the whole myocardium and septal ROI (Fig. 2). An example of the lack of congruence of the myocardium in a patient with LBBB during the different single-shot acquisitions of the T1 mapping sequence is shown in Fig. 3.

**Table 2:** Results of the T1 mapping analysis.

Parameters	Patients	Controls	Level of significance
T1_whole myocardium_ (ms)	1001 (45)	974 (29)	p = 0.006*
coefficient of variation	4.5%	3.0%	
T1_septum_ (ms)	1021 (55)	972 (25)	p = 0.002*
coefficient of variation	5.4%	2.6%	
SD T1_whole myocardium _(ms)	71 (97)	30 (48)	p = 0.014
SD T1_septum _(ms)	84 (113)	45 (61)	p = 0.058
R^2^_whole myocardium_	0.997 (0.00)	0.998 (0.00)	p = 0.008*
R^2^_septum_	0.998 (0.00)	0.999 (0.00)	p = 0.027*

T1 values are expressed as mean (SD). SD T1 and R^2^ values are expressed as median (IQR). An asterisk (*) indicates a statistically significant difference.

After manual corrections of the endo- and epicardial contours in a subanalysis, R^2^ values in patients with LBBB increased compared to semi-automated evaluation (0.997, IQR, 0.01 vs. 0.996, IQR, 0.00; p = 0.110). However, the difference was not significant and the R^2^ values of patients with LBBB remained significantly lower after manual correction compared to the control group (0.997, IQR, 0.00 vs. 0.998, IQR, 0.00; p = 0.026). More specifically, manual correction was considered necessary in all patients with R^2^ values < 0.994; in 7 out of 9 cases, R^2^ values could thus be improved. In patients with 0.994 ≤ R^2^ value ≤ 0.996, manual correction was performed in 3 out of 11 cases, leading to an improvement in 1 out of 3 cases. In all patients with R^2^ values ≥ 0.997, manual correction was not considered necessary.

Strain analysis revealed a median SDI_global_ of 0.08 (IQR 0.06) in patients with LBBB and a median SDI_global_ of 0.05 (IQR 0.01; p = 0.001) in the control group. The R^2^ values of T1 mapping showed negative correlation to SDI_global_ with ρ = − 0.376 (p = 0.008), which was considered a medium effect size^23^.

R^2^ values correlated only weakly with the QRS width, ρ = − 0.185 (p = 0.254) for the whole myocardium and ρ = − 0.232 (p = 0.150) for the septal ROI.

There was no significant group difference between patients with LBBB and apical rocking and/or septal flash and patients with LBBB without apical rocking and without septal flash in terms of R^2^ values for the whole myocardium (p = 0.235) and the septal ROI (p = 0.197).

Patients with LBBB and LGE had significantly lower R^2^ values than patients with LBBB without LGE when the whole myocardium was included in the measurement (p = 0.022). This was not the case when only the septal ROI was considered (p = 0.083). As for the clinical characteristics, a moderate inverse correlation was found between the R^2^ values and the LVEDV with ρ = − 0.299 (p = 0.020) for the whole myocardium and ρ = − 0.366 (p = 0.004) for the septal ROI and between the R^2^ values and the LVESV with ρ = − 0.335 (p = 0.009) for the whole myocardium and ρ = − 0.402 (p = 0.001) for the septal ROI.

Inter-reader agreement for all of the above parameters was good to excellent with intra-class correlation coefficients ranging from 0.876 (95% confidence interval of 0.794–0.925) and 0.989 (95% confidence interval of 0.981–0.993).

## Discussion

The present study investigated the influence of LBBB on T1 mapping. To our best knowledge, this is the first study addressing a rather common disturbance of the cardiac conduction system. Our main finding was that R^2^ values of patients with LBBB and healthy controls differed significantly, indicating a poorer goodness-of-the-T1-fit and thus a lower, yet sufficient precision in patients with LBBB^15^.

In a subgroup-analysis a moderate negative correlation of R^2^ values to global dyssynchrony of the LV could be shown by strain imaging. These findings suggest a greater variability of the goodness-of-the-fit due to asynchronous contraction of the LV in patients with LBBB compared to normal^24,25^. In patients with LBBB the relatively long acquisition window of T1 mapping due to the time of repetition might have exceeded the typical quiescent duration during diastole and therefore additionally worsened the goodness-of-the-fit. R^2^ values correlated only weakly with the QRS width, and there was no significant difference in R^2^ values between LBBB patients with and without kinetic characteristics such as septal flash or apical rocking. Therefore, thorough quality control of T1 map post-processing should be performed independently of these parameters. Manual correction of endo- and epicardial contours in patients with LBBB improved the goodness-of-the-T1 fit but the difference was not significant.

It has to be noted, that mean T1 values of patients with LBBB and healthy controls were within the scanner-specific normal range regardless of whether the whole myocardium or the septum was used for measurement. However, there was a small, but significant difference in T1 values between the patients with LBBB and the healthy controls. Various factors, such as age, gender, the presence of LBBB and pathology such as diffuse myocardial fibrosis or edema can affect T1 values and this might also be true for measured R^2^ values^26,27^.

Due to this large variety of influencing factors, we chose R^2^ analysis as an appropriate discriminator to analyze the influence of LBBB on the precision of T1 mapping since it allows an intra-individual and pixel-based assessment without the need to consider any underlying tissue alteration^15,28^.

The definitive reason for the measured differences in the goodness-of-the-fit of T1 mapping is probably multifactorial, and following aspects have to be discussed:

Asynchronous contraction in patients with LBBB might capture septum and lateral left ventricular wall in different positions during the series of single-shot acquisitions at different inversion times^3,29^. This explanation is supported by a moderate inverse correlation of R^2^ values and global dyssynchrony index. Different contraction states during the eight sequential acquisitions may also alter the influence of the partial volume effect on T1 measurements due to a varying thickness of the myocardium and out-of-plane motion^26,30–32^.

Artefacts due to respiration and patient movement may theoretically also affect the goodness-of-the-fit^31,33^. We have kept their influence as low as possible by checking datasets in advance for obvious artefacts. A scanner-side non-rigid motion correction was performed before post-processing to reduce breathing artefacts and thus no regional increases in R^2^ maps were noted^15^.

True changes in T1 values of the myocardium of patients with LBBB as a result of pathology could explain higher R^2^ values as well^34^. Although such changes cannot be ruled out in our study population, these changes were rather small and did not increase mean T1 values compared to normal values, the coefficient of variation was below the daily range of variation in both the patients with LBBB and the healthy controls when compared to the literature^35^.

Manual correction of the semi-automated evaluation tended to improve R^2^ values but not significantly, which makes the influence of the partial volume effect due to varying myocardial thickness and out-of-plane motion appear especially relevant for the precision of T1 mapping in patients with LBBB compared to the other influencing factors mentioned. It seems therefore particularly important during the semi-automated post-processing to check the forwarding of the endo- and epicardial contours as well as the septal ROIs on all single-shot acquisitions in patient with LBBB and correct them manually if necessary.

The results of our study suggest that patients with LBBB are specifically at risk of precision degradation of T1 mapping when conventional diastolic single-shot acquisition schemes are used. There are various approaches to overcome this problem. In a recent study of Liu et al., T1 mapping in systole was found to significantly improve R^2^ values in patients with mitral regurgitation and a high incidence of atrial fibrillation^19^. In LBBB the difference of myocardial contraction seems less pronounced than in atrial fibrillation, resulting in a higher R2 value in our study^19^. Systolic acquisition is associated with increased myocardial thickness, facilitating the correct placement of contours and thus reducing the influence of partial volume artefacts^27^. Systolic acquisition as proposed by Ferreira et al. in combination with a Shortened Modified Look-Locker Inversion Recovery (ShMOLLI) could therefore also be helpful in patients with LBBB, as a shorter acquisition window could reduce the influence of LBBB^31^. Other approaches that have been proposed include the acceleration of image acquisition or the use of artificial neural networks to identify and reduce motion artefacts^36,37^.

There are some limitations that need to be considered. Due to the retrospective study design, patient selection was limited; therefore, patient and control groups could not be matched for sex and age. In addition, due to the small sample size, only some subgroups could be formed to further characterize the loss of quality in T1 mapping in patients with LBBB. In particular, the influence of signal-to-noise ratio (SNR) on the mapping results could not be further evaluated here, although there is evidence that the SNR has an influence on the precision of the mapping^28,33^. It can therefore not be ruled out that, for example, differences in SNR between patients and healthy subjects also had an influence on the R^2^ values determined.

In conclusion, in patients with an LBBB, source data of T1 maps should be checked carefully for possible artefacts of the semi-automated contour detection. However, even after manual correction, a greater pixel-wise deviation from the curve model with significantly lower R^2^ values persists. In our collective, patients with LBBB did not a priori exhibit T1 values different from normal. Further studies could investigate how alternative MRI pulse sequences or different forms of post-processing can level the goodness-of-the-fit in T1 mapping between patients with and without LBBB.

## Data Availability

The datasets used and/or analyzed during the current study are available from the corresponding author on reasonable request.
